# Unawareness of a Prolonged Retained Capsule Endoscopy: The Importance of Careful Follow-Up and Cooperation between Medical Institutions

**DOI:** 10.1155/2014/909360

**Published:** 2014-09-02

**Authors:** Susumu Saigusa, Masaki Ohi, Hiroki Imaoka, Tadanobu Shimura, Ryo Uratani, Yasuhiro Inoue, Masato Kusunoki

**Affiliations:** ^1^Department of Surgery, Wakaba Hospital, 28-13 Minami-Chuo, Tsu, Mie 514-0832, Japan; ^2^Department of Gastrointestinal and Pediatric Surgery, 2-174 Edobashi, Tsu, Mie 514-8507, Japan

## Abstract

A 50-year-old man with anemia was referred to our hospital to undergo capsule endoscopy (CE), which revealed small intestinal ulcers. After 5 months of CE, he returned because of recurrent anemia without abdominal symptoms. Abdominal X-ray and computed tomography showed capsule retention in the small intestine at the pelvic cavity. The capsule remained at the same place for 7 days. We performed capsule retrieval by laparoscopy-assisted surgery with resection of the involved small intestine, including an ileal stricture. Resected specimen showed double ulcers with different morphologies, an ulcer scar with stricture, and a wide ulcer at the proximal side of the others. Each ulcer had different histopathological findings such as the degree of fibrosis and monocyte infiltration. These differences led us to consider that the proximal ulcer may have been secondarily induced by capsule retention. Our experience indicated that careful follow-up and the cooperation between medical institutions after CE examination should be undertaken for patients with incomplete examination, unknown excretion of the capsule, and/or ulcerative lesions despite the lack of abdominal symptoms. Additionally, a retained CE remaining over long periods and at the same place in the small intestine may lead to secondary ulceration.

## 1. Introduction

Capsule endoscopy (CE) is an innovative and noninvasive tool for investigating small bowel pathology. In recent years, the number of CE examination is increasing. The capsule is usually excreted with feces within 24–48 hours [1]. However, capsule retention, which is defined as having a capsule remain in the digestive tract for a minimum of 2 weeks, is known as one of the complications of CE. The rate of capsule retention has been reported to be less than 1.5% [2–4]. Capsule retention has the risk of bowel obstruction and perforation [4–9]. We report a case of CE retention for 5 months due to ileal ulcer with severe stricture without awareness.

## 2. Case Report

**Figure 1:**
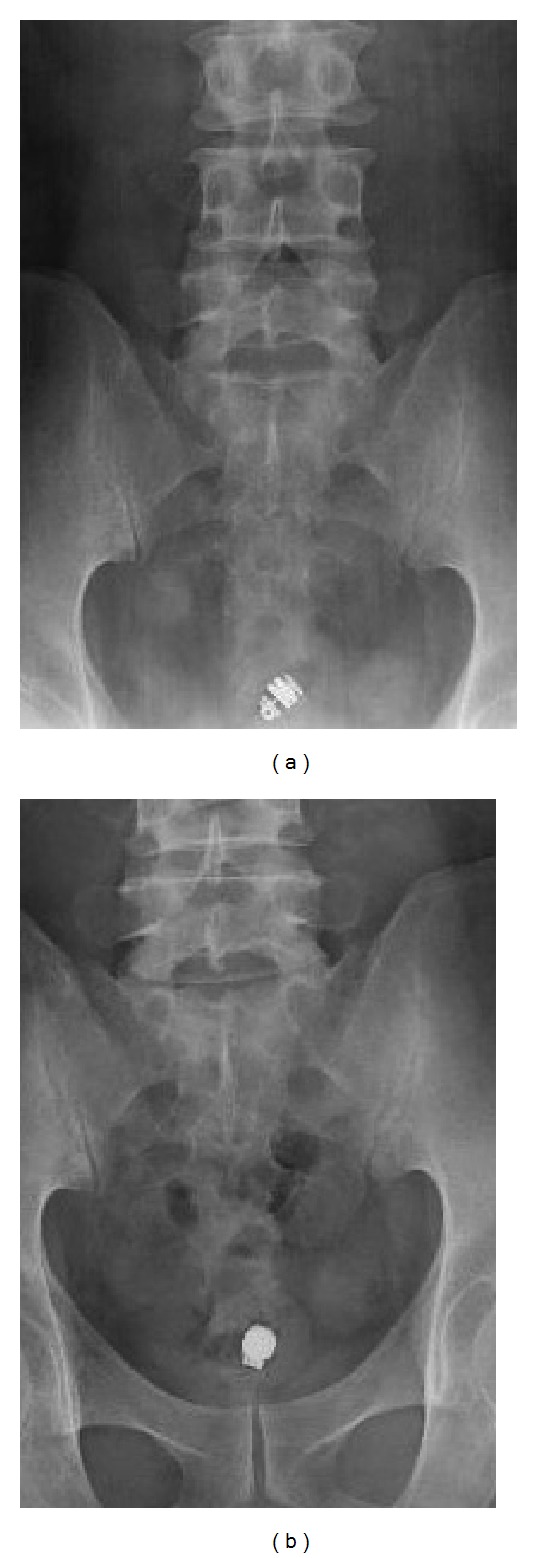
Abdominal X-ray image visualizing a CE capsule in the pelvic cavity. The capsule retention might have been overlooked if the range of the X-ray image had shifted slightly (a). Follow-up X-ray after 7 days revealed that the capsule remained at the same place (b).

**Figure 2:**
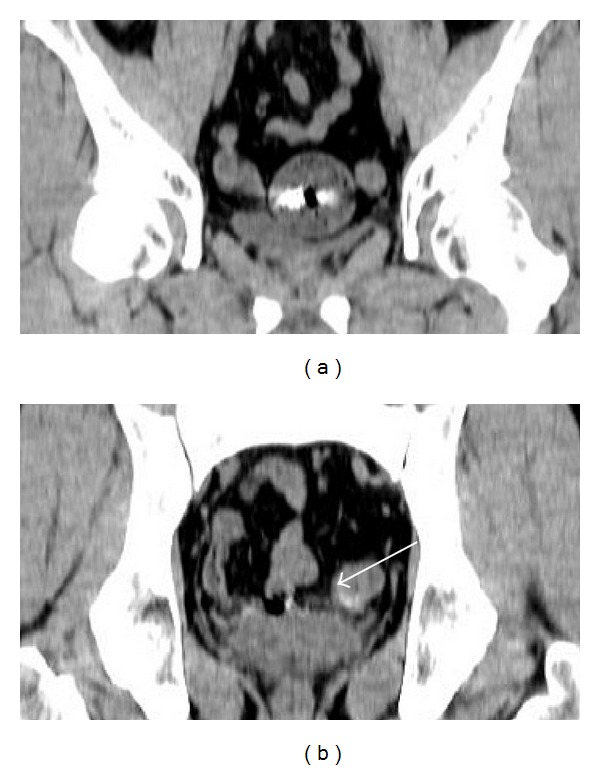
Coronal view by CT showed CE capsule retention in the small intestinal lumen with dilatation and fluid collection (a), and the finding was suspicious for stenosis ((b), arrow).

**Figure 3:**
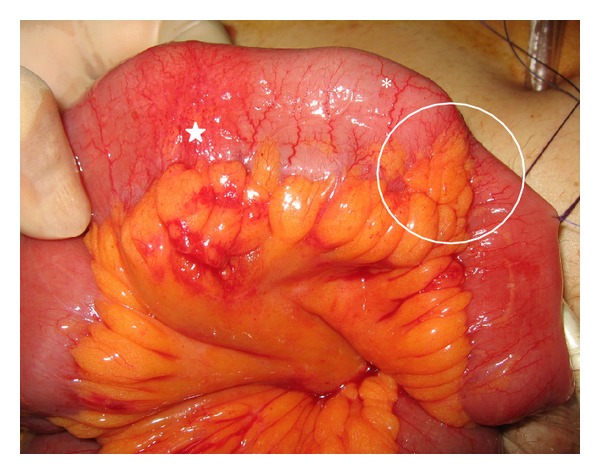
Operative findings. Retained CE capsule (∗). Reddened serosa (star). Stenosis with the wrapping-sign and caliber change (inside the circle).

**Figure 4:**
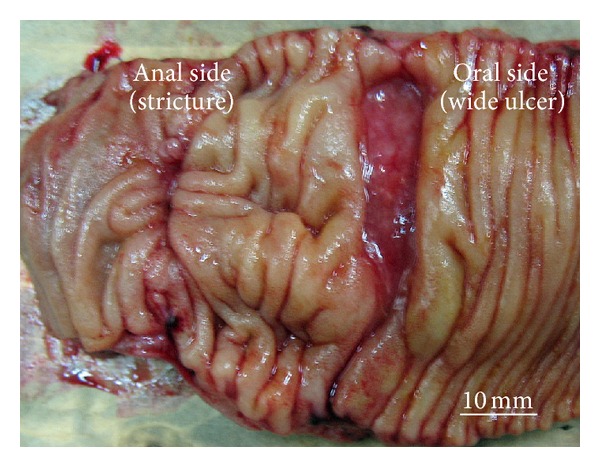
Resected lesion showed double ulcers. There was a wide ulcer at the proximal side of the ulcer scar.

**Figure 5:**
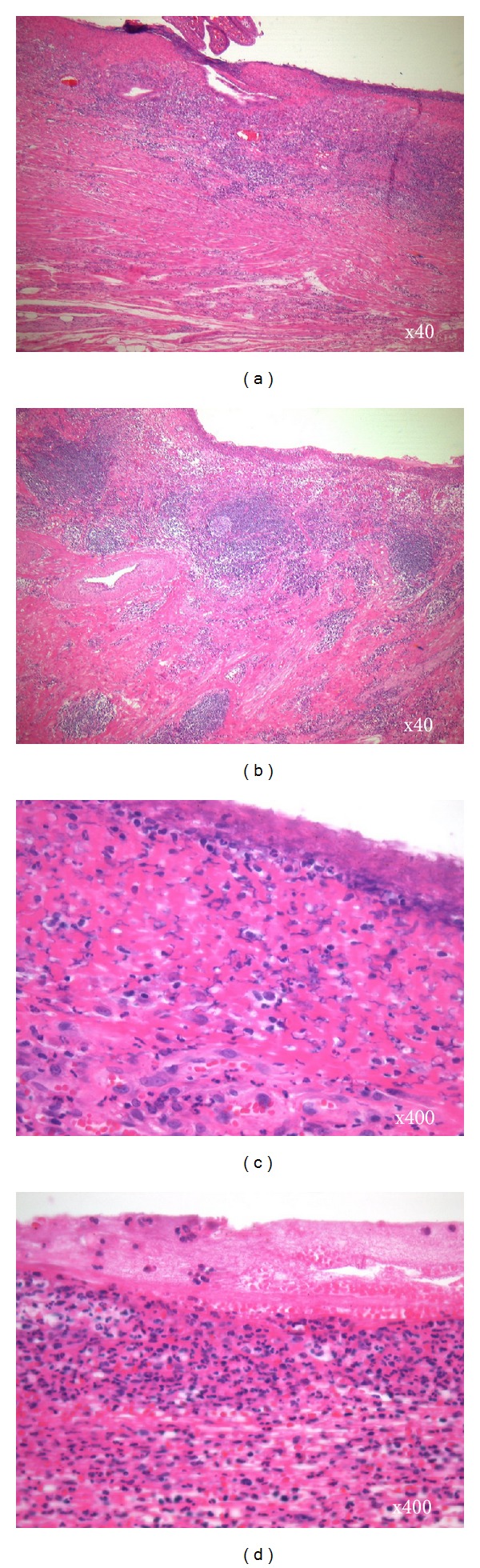
Histopathological examination revealed that an ulcer scar at the distal side had mainly fibrosis ((a) and (c)), while lymphoid follicles, infiltration of monocytes, and neutrophils were more noticeable in the wide ulcer on the proximal side ((b) and (d)). Hematoxylin-eosin staining. Original magnification 40x: (a) and (b); 400x: (c) and (d).

A 50-year-old man with gradually worsening anemia and suspected small bowel bleeding was referred to our hospital to undergo CE because esophagogastroduodenoscopy and total colonoscopy did not reveal the source of the gastrointestinal bleeding. His oral medications included several psychoactive drugs and iron preparations but not nonsteroidal anti-inflammatory drugs. He had no history of abdominal surgery. We did not perform patency examination before CE because we assessed low possibility of severe stenosis and inflammatory bowel disease based upon his clinical history and abdominal X-ray examination. CE (PillCam SB2 system, Given Imaging, Yokneam, Israel) demonstrated several ulcers at the small intestine, but the capsule did not reach the cecum during the recording time (stomach transit time: 220 minutes). These results were sent to his primary care doctor. Five months after the CE examination, he was referred again for the recurrence of anemia. Abdominal X-ray examination revealed that the capsule was retained at the pelvic cavity (Figure 1(a)). Follow-up abdominal X-ray examination after 7 days demonstrated that the capsule remained in exactly the same part but was rotatable (Figure 1(b)). Computed tomography (CT) showed that the capsule seemed to be floating in the small intestinal lumen with dilatation and fluid collection proximal to the capsule (Figure 2(a)). Additionally, the CT coronal view indicated findings suspicious for stenosis distal to the capsule (Figure 2(b)). The patient had had regular bowel movements and no abdominal complaints for the past 5 months. He could not confirm that the capsule was egested because he failed to monitor his stools. After proper informed consent was obtained, we retrieved the capsule by laparoscopy-assisted surgery. The patient declined endoscopic approach and treatments. Laparoscopic instruments were placed through 3 trocars. The CE was laparoscopically detected approximately 50 cm from the end of the ileum. After fluoroscopic confirmation, the part of the ileum with the capsule was moved outside the abdominal cavity, and we made the following observations around the area of retention: the fat-wrapping sign and a caliber change were observed distal to the capsule, with reddened serosa proximal to the capsule. The capsule could not pass through this stricture (Figure 3). The small intestine was extensively evaluated, and no other abnormalities were found. We resected approximately 30 cm of ileum and performed a functional end-to-end anastomosis. An ulcer scar with stricture was macroscopically observed, and a wide ulcer was observed at the proximal side of the lesion (Figure 4). On histopathological examination, the lesions were determined to be a nonspecific ulcer (Ul III) with fibrosis, formation of lymphoid follicles, and infiltration of monocytes and neutrophils to the subserosa, without evidence of malignancy, inflammatory bowel disease, or tuberculosis (Figure 5). The patient's postoperative course was uneventful, and he was discharged on the postoperative day 8. There was no evidence of progression of anemia at a follow-up visit conducted 7 months after surgery.

## 3. Discussion

An incomplete examination means that there is failure of the capsule to reach the cecum during the recording time. It has been reported that the rate of incomplete examination ranges from 16.5 to 20% [3, 4]. In our case, the patient had an incomplete examination and subsequently failed to determine if the capsule was egested. Moreover, his primary care doctor did not perform an X-ray examination because the patient remained asymptomatic for 5 months after CE examination despite a severe ileal stricture caused by a simple ulcer. It has been reported that most patients with CE retention remain asymptomatic [2–4, 10, 11]. Our case indicated that follow-up X-ray examination after CE should routinely be performed for patients with incomplete examination and unknown capsule excretion despite a lack of abdominal symptoms, and the cooperation between medical institutions is essential.

Although there were many reports of capsule retention of long duration [4, 12, 13], we think that the retained CE should be retrieved if spontaneous or pharmaceutical manipulation, the rates of spontaneous or pharmaceutical-manipulated passage of retained capsules have been reported to vary from 15 to 65.6% [3, 14], is ineffective to egest it because there are several reports of retained capsule causing intestinal obstruction and perforation [5, 6, 8, 9]. Surgical retrieval is often required secondary to an underlying pathologic process causing a stricture or obstruction although double-balloon endoscopy has been reported as one of the effective approaches for the retrieval of retained CE [15, 16]. We removed the retained CE by laparoscopy-assisted surgery. Laparoscopic detection of the retained CE and the intestinal abnormality were easy and useful. Moreover, definitive surgery to resect the culprit stricture was performed. Except in cases of known or suspected Crohn's disease, laparoscopy-assisted surgery may be the first choice for the retrieval of retained CE in the small intestine because this procedure can simultaneously allow for diagnosis and treatment.

In the current case, histopathological examination revealed double ulcers of nonspecific cause without evidence of malignancies, Crohn's disease, or tuberculosis. A comparison of the 2 ulcers histopathologically found that an ulcer scar at the distal side had mainly fibrosis (Figures 5(a) and 5(c)), while lymphoid follicles and monocyte infiltration were more noticeable in the wide ulcer on the proximal side (Figures 5(b) and 5(d)). These differences in the degree of inflammation led us to suspect that the wide ulcer at the proximal side of the stricture may have been secondarily induced by the capsule retention and resulted in the recurrence of anemia. However, our verifications are insufficient to clarify whether the proximal ulcer is truly caused by capsule retention because we could not detect the multinucleate giant cells, which is known as the foreign body response [17, 18], in the wide ulcer on the proximal side.

In conclusion, our experience indicated that careful follow-up after CE examination should be undertaken for patients with incomplete examination, unknown excretion of the capsule, and/or ulcerative lesions despite the lack of abdominal symptoms, and the cooperation between medical institutions is essential with increasing CE examination. Additionally, a prolonged CE retention in the same part of the small intestine should be retrieved because it may further endanger the patient with more “ammunition” for secondary ulceration and perforation.

## Consent

Written informed consent was obtained from the patient for publication of this case report and accompanying images.
